# Viperin deficiency promotes dendritic cell activation and function via NF-kappaB activation during *Mycobacterium tuberculosis* infection

**DOI:** 10.1007/s00011-022-01638-3

**Published:** 2022-10-31

**Authors:** Xinying Zhou, Hui Xu, Qianna Li, Qi Wang, Honglin Liu, Yingqi Huang, Yao Liang, Linmiao Lie, Zhenyu Han, Yaoxin Chen, Yulan Huang, Wenle Zhou, Qian Wen, Chaoying Zhou, Shengfeng Hu, Li Ma

**Affiliations:** Institute of Molecular Immunology, School of Laboratory Medicine and Biotechnology, Southern Medical University, Guangzhou, 510515 China

**Keywords:** Viperin, Dendritic cells (DCs), *Mycobacterium tuberculosis* (Mtb), NF-kappaB, Immune responses

## Abstract

**Objectives and design:**

Dendritic cells (DCs) are one of the key immune cells in bridging innate and adaptive immune response against *Mycobacterium tuberculosis* (Mtb) infection. Interferons (IFNs) play important roles in regulating DC activation and function. Virus-inhibitory protein, endoplasmic reticulum-associated, interferon-inducible (Viperin) is one of the important IFN-stimulated genes (ISGs), and elicits host defense against infection.

**Methods:**

We investigated the effects and mechanisms of Viperin on DC activation and function using Viperin deficient bone marrow-derived dendritic cells (BMDCs) during Mtb infection.

**Results:**

Viperin deficiency enhanced phagocytic activity and increased clearance of Mtb in DCs, produced higher abundance of NO, cytokine including interleukin-12 (IL-12), Tumor necrosis factor-α (TNF-*α*), IL-1*β*, IL-6 and chemokine including CXCL1, CXCL2 and CXCL10, elevated MHC I, MHC II and co-stimulatory molecules expression, and enhanced CD4^+^ and CD8^+^ T cell responses. Mechanistically, Viperin deficiency promoted DC activation and function through NF-κB p65 activation. NF-κB p65 inhibitor prevented cytokine and chemokine production, and co-stimulatory molecules expression promoted by Viperin deficiency.

**Conclusions:**

These results suggest that Mtb induced Viperin expression could impair the activation of host defense function of DCs and DC-T cell cross talk during Mtb infection. This research may provide a potential target for future HDT in TB therapy.

**Supplementary Information:**

The online version contains supplementary material available at 10.1007/s00011-022-01638-3.

## Background

Tuberculosis (TB) caused by *Mycobacterium tuberculosis* (Mtb) infection is one of the leading killers among chronic infectious diseases worldwide. It has been reported that 9.9 million new TB cases and 1.5 million TB deaths in 2020, and deaths from TB have risen for the first time within 15 years [1]. Although the standard therapy known as Directly Observed Treatment, Short course (DOTS) is effective for treating drug-sensitive TB, lengthy duration of treatment and drug-induced tissue toxicity, contributes to patient non-compliance and drug resistance toward Mtb [2]. Hence, it is imperative to improve current or develop new TB treatment strategies. As a long-term evolved pathogen, Mtb is capable to evade both innate and adaptive immunity. Therefore, host-directed therapy (HDT) as an emerging concept, which can modulate host immune response with small molecules, holds the promising to avoid drug resistance and achieve efficiency for anti-TB treatment.

Dendritic cells (DCs) are professional antigen-presenting cells (APCs) linking innate and adaptive immune responses, and key component of the host defense system during Mtb infection [3]. Immune response of DCs to Mtb infection is initiated following the uptake of Mtb by phagocytes [4]. Reactive oxygen/nitrogen species and NO production are produced and play critical roles in determining the fate of intracellular Mtb within phagocytes [5]. The interaction between phagocyte pattern recognition receptors (PRRs) and Mtb antigens triggers the production of various proinflammatory cytokines, including interleukin-12 (IL-12), tumor necrosis factor-α (TNF-α), IL-1β and IL-6, as well as chemokines that recruit and activate other innate and adaptive immune cells from the circulation to the site of infection. Major histocompatibility complex class I (MHC I), MHC II and co-stimulatory molecules, including CD80, CD86 and CD40, which are upregulated by Mtb infection, directly modulate the function of DCs in antigen presentation and T-cell activation [6, 7]. These biological functions of DCs trigger a range of cellular events that are capable of killing Mtb. However, Mtb has evolved numerous evasion mechanisms to hijack innate immune response for immune escape, leading to the chronic infection [8]. Taken together, the intracellular survival of Mtb is regarded as a compromise outcome of the host immune response and the resistance of Mtb. Hence, strengthening the immune responses of DCs in combating Mtb through HDT could be one of the potential approaches for effective bacterial killing and clearance of infection/disease.

Interferons (IFNs) therapy leads to beneficial or detrimental outcomes against TB, which is highly context dependent on different settings of host-bacteria encounters [9, 10]. This discrepancy might be due to hundreds of thousands of IFN-stimulated genes (ISGs) activated by IFNs with various functions. Virus-inhibitory protein, endoplasmic reticulum-associated, interferon-inducible (Viperin) encoded by *Rsad2* is one of the important ISGs that participates in both innate and adaptive immune system in macrophages, DCs and T cells. It can interact with a large number of viral and host proteins to elicit host immune responses against a wide spectrum of viruses [11–13]. Viperin can be stimulated by a variety of stimulations such as IFNs, dsRNA, LPS or viruses [11]. However, the effects and mechanisms of Viperin on regulating DCs maturation and function during Mtb infection remain unknown.

In our study, we found that Viperin was up-regulated by Mtb infection. Viperin deficiency promoted phagocytosis and enhanced clearance of Mtb in DCs. Furthermore, Viperin deficiency significantly increased NO production, cytokine production including IL-12, TNF-α, IL-1β and IL-6, as well as chemokine production including CXCL1, CXCL2 and CXCL10. In addition, Viperin deficiency increased expression of major histocompatibility complex class I (MHC I), MHC II and co-stimulatory molecules, and enhanced DC functions in promoting CD4^+^ and CD8^+^ T-cell activation. Thus, we propose that Viperin suppresses DC functions and impedes the robust CD4^+^ and CD8^+^ T cell responses, which attenuate host immune surveillance, resulting in immune escape of Mtb. Thus, Viperin deficiency could boost the defense function of DCs, and we propose it constitutes a potential target for future HDT in TB therapy.

## Methods

### Ethics statement

This study was approved by the Ethics Committee of Southern Medical University. The Animal ethical certification and animal handling procedures were approved by the Animal Experimental Center in Southern Medical University. All experimental protocols were reviewed and approved by the Medical Ethics Board and the Biosafety Management Committee of Southern Medical University. Highly pathogenic microorganism laboratory management commitment letter was approved by Southern Medical University.

### Mice

C57BL/6J mice were purchased from the Lab Animal Center of Southern Medical University (Guangzhou, China). Viperin deficient (*Rsad2*^*−/−*^) mice on a C57BL/6J background were built by Nanjing Biomedical Research Institute (Nanjing, China). OT-I mice were obtained from the Pathology Laboratory of Southern Medical University. OT-II mice were obtained from Fujian Medical University. All mice were maintained in the Lab Animal Center of Southern Medicine University under specific pathogen-free conditions.

### Mtb culture and infection

Mtb standard strain H37Rv (American Type Culture Collection) was grown in 7H9 (Becton Dickinson, New Jersey, USA) broth containing 0.2% glycerol (GHTECH, Guangzhou, China) and 10% OADC at 37 °C in 5% CO_2_, OADC consists of 0.06% volume oleic acid (SIGMA, St. Louis, MO, USA), 5% albumin (SIGMA, St. Louis, MO, USA), 100 mM glucose (GHTECH, Guangzhou, China), 0.003% catalase (SIGMA, St. Louis, MO, USA), 145 mM NaCl (GHTECH, Guangzhou, China). Ground the agglomerated H37Rv into a bacterial suspension with cell culture medium or PBS and measured the absorbance at 600 nm. After the concentration was calculated, the cells were infected according to the multiplicity of infection (MOI) required by the experiment.

### Cell culture

For generation of bone marrow-derived dendritic cells (BMDCs) from *Rsad2*^+/+^ and *Rsad2*^−/−^ mice, bone marrow cells from femurs and tibiae was flushed with RPMI-1640 medium (Corning, NY, USA), cultured in RPMI-1640 containing 10% FBS (Corning, NY, USA) and mGM-CSF (20 ng/ml; Pepro Tech, USA) at 37 °C in 5% CO_2_ after lysing red blood cells. Fresh medium (10 ml) containing the same ingredient was provided to the culture on day 3. At day 6, half of the volume (10 ml) was removed and centrifuged the cells at 1600 rpm for 5 min. Cells were resuspended with the same volume and ingredients of fresh medium and cultured in the Petri dishes. BMDCs were used on day 8, cell culture supernatants were removed and centrifuged at 1400 rpm for 5 min. Cells were resuspended in fresh medium containing 2 ng/ml of mGM-CSF and seeded in cell culture plates for further experiments.

### Treatment of inhibitors in BMDCs

BMDCs were pretreated with NF-kappaB (NF-κB) p65 inhibitor JSH-23 (20 µM; Selleckchem, Houston, USA) or iNOS inhibitor L-NAME HCl (1 mM; Selleckchem, Houston, USA) for 1 h before Mtb infection.

### RNA extraction and quantitative real time PCR (qRT-PCR)

Total cellular RNA was extracted by Trizol method, and RNA concentration was measured by NanoDrop 2000 (Thermo Fisher Scientific, Carlsbad, CA, USA). RNA was reverse transcribed into cDNA using HonorTM II 1st Strand cDNA Synthesis SuperMix (Novogene, Beijing, China) after removal of genomic DNA (gDNA). CDNA was added to a mixture of Unique Aptamer qPCR SYBR Green Master Mix (Novogene, Beijing, China) and primers (Sangon Biotech, Shanghai, China) to detect the expression of related genes on a LightCycler 480 thermocycler (Roche, Basel, Switzerland). A three-step reaction method was used to perform qPCR, after a 2-min preincubation at 95 °C, target genes were amplified and quantified (95 °C for 15 s, 65 °C for 15 s, 68 °C for 20 s) for 45 cycles, followed by a 30 s cooling at 37 °C. All PCR products were normalized using GAPDH as an internal control, the expression of individual gene was calculated using the 2^−△△CT^ method and expressed as fold change. The complete mouse primers are GAPDH (F: CATCACTGCCACCCAGAAGACTG, R: ATGCCAGTGAGCTTCCCGTTCAG); Rsad2 (F: GGAAGGTTTTCCAGTGCCTCCT, R: ACAGGACACCTCTTTGTGACGC); IL-12p40 (F: TTGAACTGGCGTTGGAAGCACG, R: CCACCTGTGAGTTCTTCAAAGGC); TNF (F: GGTGCCTATGTCTCAGCCTCTT, R: GCCATAGAACTGATGAGAGGGAG); IL-1β (F: TGGACCTTCCAGGATGAGGACA, R: GTTCATCTCGGAGCCTGTAGTG); IL-6 (F: TACCACTTCACAAGTCGGAGGC, R: CTGCAAGTGCATCATCGTTGTTC); iNOS (F: GAGACAGGGAAGTCTGAAGCAC, R: CCAGCAGTAGTTGCTCCTCTTC); CXCL1 (F: TCCAGAGCTTGAAGGTGTTGCC, R: AACCAAGGGAGCTTCAGGGTCA); CXCL2 (F: CATCCAGAGCTTGAGTGTGACG, R: GGCTTCAGGGTCAAGGCAAACT); CXCL10 (F: ATCATCCCTGCGAGCCTATCCT, R: GACCTTTTTTGGCTAAACGCTTTC); SLC2α1 (F: GCTTCTCCAACTGGACCTCAAAC, R: ACGAGGAGCACCGTGAAGATGA); PFKFB3 (F: TCATCGAGTCGGTCTGTGACGA, R: CATGGCTTCTGCTGAGTTGCAG); HK2 (F: CCCTGTGAAGATGTTGCCCACT, R: CCTTCGCTTGCCATTACGCACG); PDK1 (F: CCACTGAGGAAGATCGACAGAC, R: AGAGGCGTGATATGGGCAATCC); HIF-1α (F: CCTGCACTGAATCAAGAGGTTGC, R: CCATCAGAAGGACTTGCTGGCT); TKT (F: GCTAACATCCGAATGCCTACGC, R: T TGGTGTCTCCATCCAGGGCAA).

### Protein sample preparation and western blot analysis

Cells were harvested and washed twice with ice-cold PBS, and then collected in ice-cold lysis buffer containing 455 mM Tris HCl (pH 6.8) (Sangon Biotech, Shanghai, China), 41.6 mM SDS (Zhuosheng Biotech, Shanghai, China), 26.9 μM bromophenol blue (Solarbio), 30% (v/v) glycerol (SIGMA, St. Louis, MO, USA), and 10 μM DL-Dithiothreitol (DTT) (SIGMA, St. Louis, MO, USA). They were heated at 95 °C for 10 min to fully denature the protein. Equal amounts of cell lysates were loaded into 8–15% polyacrylamide gels. After electrophoresis, proteins were electric transferred onto a polyvinylidene difluoride (PVDF) membrane (Merck KGaA, Germany). Membranes were blocked in 5% (w/v) BSA (SIGMA, St. Louis, MO, USA) in PBST for 1 h at room temperature and incubated overnight with primary antibodies at 4 °C. The membranes were washed three times with PBST for 10 min each time, and incubated with HRP-conjugated goat anti-rabbit or goat anti-mouse secondary antibodies (Thermo Fisher Scientific, Carlsbad, CA, USA) for 1 h at room temperature. After washing the membranes three times with PBST for 10 min each time, the immunoblots were visualized with Immobilon Western Chemiluminescence HRP substrate (ECL; Thermo Fisher Scientific, USA) on FluorChem Systems (ProteinSimple, USA) according to the manufacturer’s protocol. The integrated density of all protein bands were analyzed by Image J software (National Institutes of Health) and normalized to β-Actin or GAPDH. All the antibodies used in this project are listed in Supplemental Table 1.

### Phagocytosis assay

BMDCs were seeded in 12-well cell culture plates (CELLTER, China) at the 5 × 10^5^ cells per well for 24 h. Cells were harvested and incubated with OVA-FITC (Bohu Biotec, Shanghai, China) and fluorescently conjugated mAb CD11c^+^ at 37 ℃ in 5% CO_2_ for 30 min, or collected after infected with Texas Red (SIGMA, St. Louis, MO, USA) tagged H37Rv for 2 h at MOI = 5 and incubated with mAb CD11c^+^ at 4 °C for 30 min. The whole process needs to be protected from light. Then we analyzed the percentage of positive cells and relative median fluorescence intensity (MFI) in Texas-Red and FITC by flow cytometry and cells were gated on CD11c^+^.

### Flow cytometry analysis

Cells were centrifuged at 1600 rpm for 5 min and washed once with 1% BSA/PBS. Then cells were incubated with mixtures of fluorescently conjugated mAbs for 30 min at 4 °C and washes twice with 1% BSA/PBS. Cell phenotypes were analyzed by flow cytometry on BD LSR II Fortessa X-20 (BD Biosciences, USA). Data were acquired as the fraction of labeled cells or MFI within a live-cell gate using FlowJo software. All gates were set on the basis of isotype-matched control antibodies. MAbs of mice were as follows: V450-anti-MHC I, PE-Cy7-anti-MHC II, V450-anti-CD40 (eBioscience, Carlsbad, CA, USA), FITC-anti-CD80, APC-anti-CD86, FITC-anti-CD3, PerCP-Cy5.5-anti-CD4^+^, APC-anti-CD8^+^, PE-Cy7-CD69 (TONBO Bioscience, San Diego, CA, USA).

### Colony-forming units (CFU) assay

BMDCs were seeded in 12-well plates at 5 × 10^5^ cells per well for 24 h and cells were infected with H37Rv at MOI of 5 at 37 °C in 5% CO_2_ for 1 h. To remove the extracellular H37Rv, the cells were thoroughly washed three times with PBS. The cells used to detect the amount of phagocytosis were lysed and diluted with 0.01% TritonX-100 (Solarbio, Beijing, China). 50 μl of the dilution was evenly spread on the 7H10 agar plates (Becton Dickinson, New Jersey, USA) supplemented with 10% OADC. Other cells were incubated in RPMI-1640 containing 10% FBS and mGM-CSF (2 ng/ml) at 37 °C in 5% CO_2_ for 48 and 72 h later. Then, they were lysed and diluted and plated on 7H10 agar plates in the same way. All plates were placed upside down at 37 °C in 5% CO_2_ and counted after colony growth.

### Enzyme-linked immunosorbent assay (ELISA)

Cell culture supernatant was collected and filtered through a 0.22 μm filter to remove cell debris and Mtb. The secretion of cytokines (IL-12p70, TNF-α, IL-1β and IL-6) and chemokines (CXCL1, CXCL2, CXCL10, CCL3 and CCL4) in the supernatant was analyzed by respective ELISA kit (MultiSciences, Hangzhou, China). All procedures were performed according to the manufacturer’s instructions. Absorbance at 450 nm and 630 nm was detected by a Microplate Reader (TECAN SPARK, Austria).

### Assay of cholesterol

Cholesterol content of BMDCs was detected with the Amplite™ Cholesterol Quantitation Kit (AAT Bioquest, Sunnyvale, CA, USA). BMDCs were seeded at 5 × 10^5^ cells per well of 12 wells plate and infected with H37Rv at MOI = 2 for 24 h. Cells were lysed with 1 ml of 0.1% TritonX-200 per well after washing three times with PBS. Reagents were prepared according to the manufacturer’s protocol. 50 μl lysates were layouted and standard in a solid black 96-well microplate. Then, 50 μl cholesterol assay working solution was added into each well and cells were incubated at 37 °C for 30 min, protected from light. Fluorescence intensity was monitored at Ex/Em = 540/590 nm using a Microplate Reader (TECAN SPARK, Austria).

### Nitric oxide (NO) measurement

Griess-Reagent System (Promega, Madison, USA) was used to detect NO production of BMDCs. 50 μlstandard diluted as instructed and 50 μl cell supernatant were added in a round-bottom 96-well plate. Then, 50 μl sulfanilamide solution was added and incubated them for 5–10 min at room temperature, protected from light. 50 μl NED solution was added to all wells and the reaction was incubated at room temperature for another 5–10 min, protected from light. Absorbance was measured within 30 min with a Microplate Reader (TECAN SPARK, Austria) between 520 and 550 nm. NO concentration was calculated according to the standard curve.

### Antigen presentation assays

BMDCs were seeded at 1 × 10^6^ cells per well in 6 wells plate (CELLTER, China). CD4^+^ T cells from OT-II mice or CD8^+^ T cells from OT-I mice were purified by CD4^+^ or CD8^+^ microbeads (Miltenyi Biotec, Germany). To detect cell proliferation, cells were labeled with CFSE (2 μM; Selleckchem, Houston, USA) at 37 °C for 20 min. To detect cell activation, cells were resuspended with RPMI-1640 containing 10% FBS and IL-2 (20 ng/ml) and seeded at 5 × 10^4^ cells per well in round-bottomed 96-well plates. The next day, BMDCs were incubated with OVA (1 mg/ml; Bohu Biotec, Shanghai, China) and OVA_323–339_ (1 μg/ml; Sangon Biotech, Shanghai, China) or OVA_257–264_ (1 μg/ml; Sangon Biotech, Shanghai, China) for 6 h, washed twice with RPMI-1640 containing 10% FBS and IL-2 (20 ng/ml; Pepro Tech, USA) and co-cultured with purified T cells at a ratio of DC: T cell = 1: 10 for 24 h, flow cytometry analysis of the expression of activation index CD69. For 72 h, proliferation was determined by detecting the fluorescence intensity of CFSE via flow cytometry.

### Statistical analysis

Data are expressed as mean ± SD at least three independent experiments. Determination of statistical differences was performed with Graphpad Prism 6.0 Software using Student t-test. *p ≤ 0.05, **p ≤ 0.01 were considered as statistically significant.

## Results

### Mtb infection promotes viperin expression in DCs

**Fig. 1:**
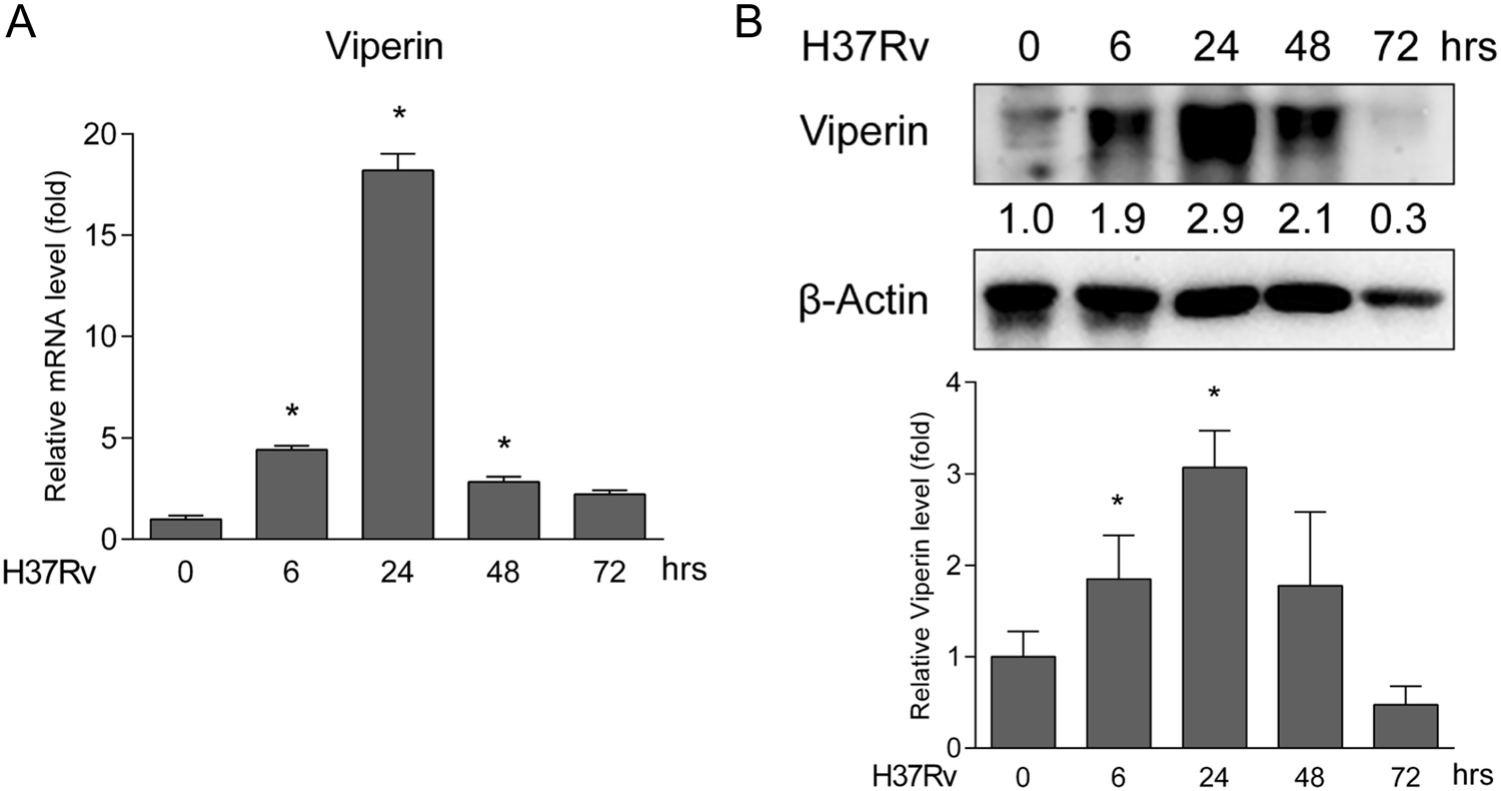
Mtb infection promotes Viperin expression in DCs. **A** Viperin mRNA and **B** protein expression were detected by qRT-PCR and Western blot assay in BMDCs at 6, 24, 48 and 72 h.p.i. **A** Data are presented as mRNA fold change relative to uninfected controls and at least *n* = 3 independent experiments with each 2 replicates are shown. Data shown are the mean ± SD, *T*-test, *p ≤ 0.05. **B** Densitometric analysis was performed for Western blot analysis. β-Actin served as an internal reference. The numbers below immunoblot indicates the density ratio of Viperin/β-Actin. Data are presented as fold change relative to uninfected control and are representative of three independent experiments with similar results. The ratio of protein expression of Viperin is shown in graph. Data shown are the mean ± SD, *T*-test, *p ≤ 0.05

BMDCs were infected with H37Rv at different time points and Viperin expression levels were determined. Viperin mRNA expression significantly increased at 6, 24 and 48 h and the peak expression was induced at 24 h after Mtb infection (Fig. 1A). Viperin protein expression was significantly induced at 6, 24 and 48 h, and the peak expression was at 24 h (Fig. 1B). These results indicate that Viperin expression increased by Mtb infection may play a key role in regulating immune response in DCs.

### Viperin deficiency promotes phagocytic function of DCs and suppresses Mtb clearance

**Fig. 2:**
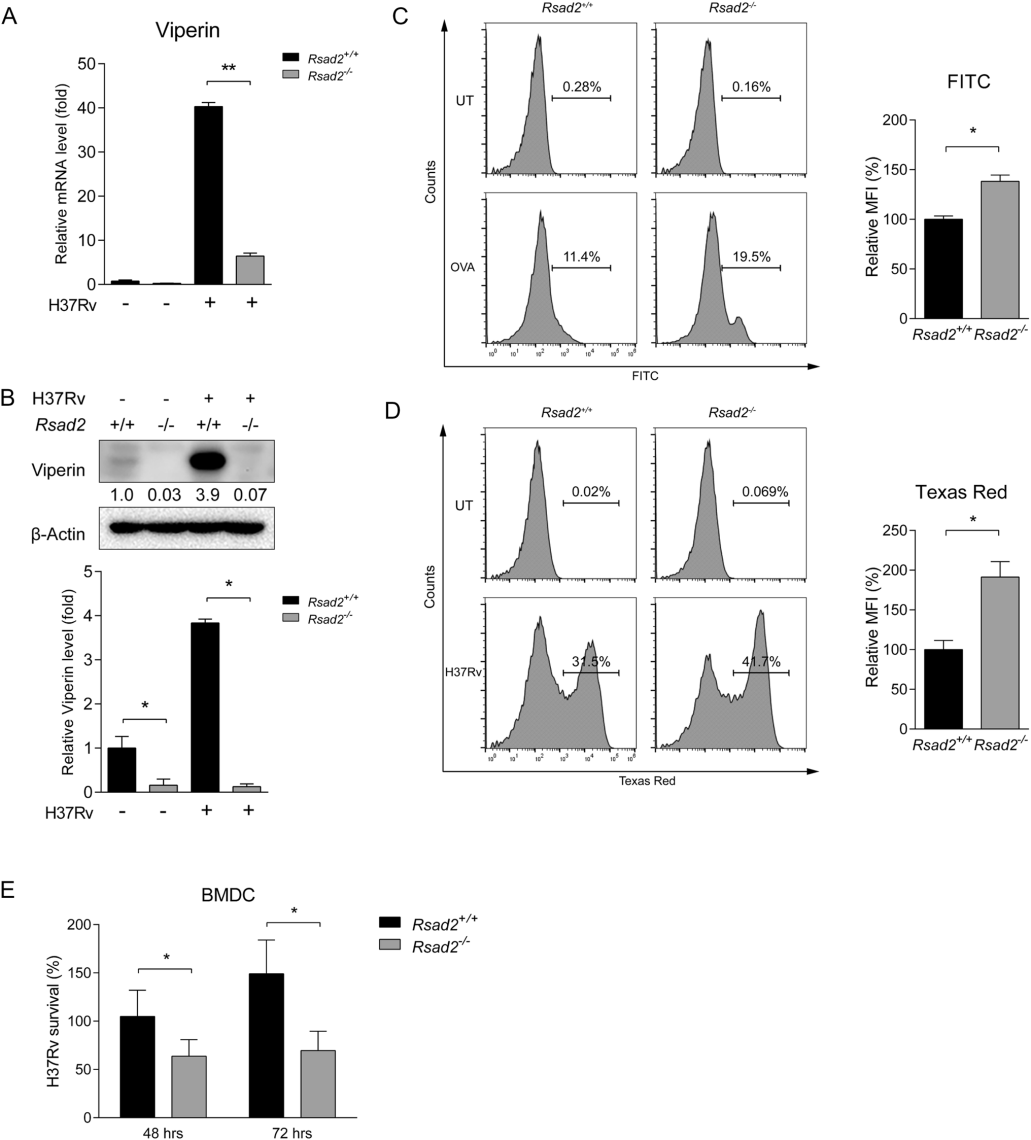
Viperin deficiency promotes phagocytic function of DCs and facilitates Mtb clearance. **A** Viperin mRNA and **B** protein expression were detected by qRT-PCR and Western blot analysis in *Rsad2*^*−/−*^ BMDCs with H37Rv infection at MOI = 2 for 24 h. **C** BMDCs were incubated with OVA-FITC and fluorescently conjugated mAb CD11c^+^ at 37 °C in 5% CO_2_ for 30 min. Percentage of FITC positive BMDCs (CD11c^+^) and MFI was analyzed by flow cytometry. **D** BMDCs were infected with Texas Red tagged H37Rv for 2 h at MOI = 5. Cells were collected and incubated with fluorescently conjugated mAb CD11c^+^ at 4 °C for 30 min. Percentage of Texas Red positive BMDCs (CD11c^+^) and MFI were analyzed by flow cytometry. **E** C.f.u assay was performed to analyze H37Rv survival (MOI = 5) in *Rsad2*^*−/−*^ BMDCs at 48 and 72 h.p.i. **A** Data are presented as mRNA fold change relative to uninfected control and at least *n* = 3 independent experiments with each 3 replicates are shown. **B** Densitometric analysis was performed after Western blot analysis. β-Actin served as an internal reference. The numbers below immunoblot indicate the density ratio of Viperin/β-Actin. Data are presented as fold change relative to uninfected control of *Rsad2*^+*/*+^ BMDCs and are representative of three independent experiments with similar results. The ratio of protein expression of Viperin is shown in graph. **C**, **D** Data are presented with *Rsad2*^+*/*+^ BMDCs as control 100% and are representative of 3 independent experiments with each 4 replicates. **E** Data are presented with the 0 h Mtb counts control of *Rsad2*^+*/*+^ BMDCs is used as 100% and are representative of 3 independent experiments with each 4 replicates. **A**–**E** Data shown are the mean ± SD, *T*-test, *p ≤ 0.05, **p ≤ 0.01

We applied Viperin-deficient (*Rsad2*^−/−^) mice to investigate the role of Viperin in regulating phagocytic function of DCs. Neither mRNA nor protein expression of Viperin was detected in *Rsad2*^−/−^ BMDCs with Mtb infection for 24 h, indicating successful disruption of *Rsad2* in BMDCs (Fig. 2A and B). Next, BMDCs phagocytic OVA-FITC and H37Rv with Texas Red were detected by flow cytometry. The results showed that Viperin deficiency significantly promoted amounts of OVA and H37Rv within DCs, indicating that Viperin inhibited the phagocytic function of DCs (Fig. 2C and D). Moreover, we found that Viperin deficiency led to significant decrease of Mtb infection at 48 and 72 h in DCs detected by CFU assay (Fig. 2E). These results indicate that Viperin inhibits phagocytic function of DCs and increases Mtb infection in DCs.

### Viperin deficiency elicits robust cytokine, chemokine and NO production in DCs

**Fig. 3:**
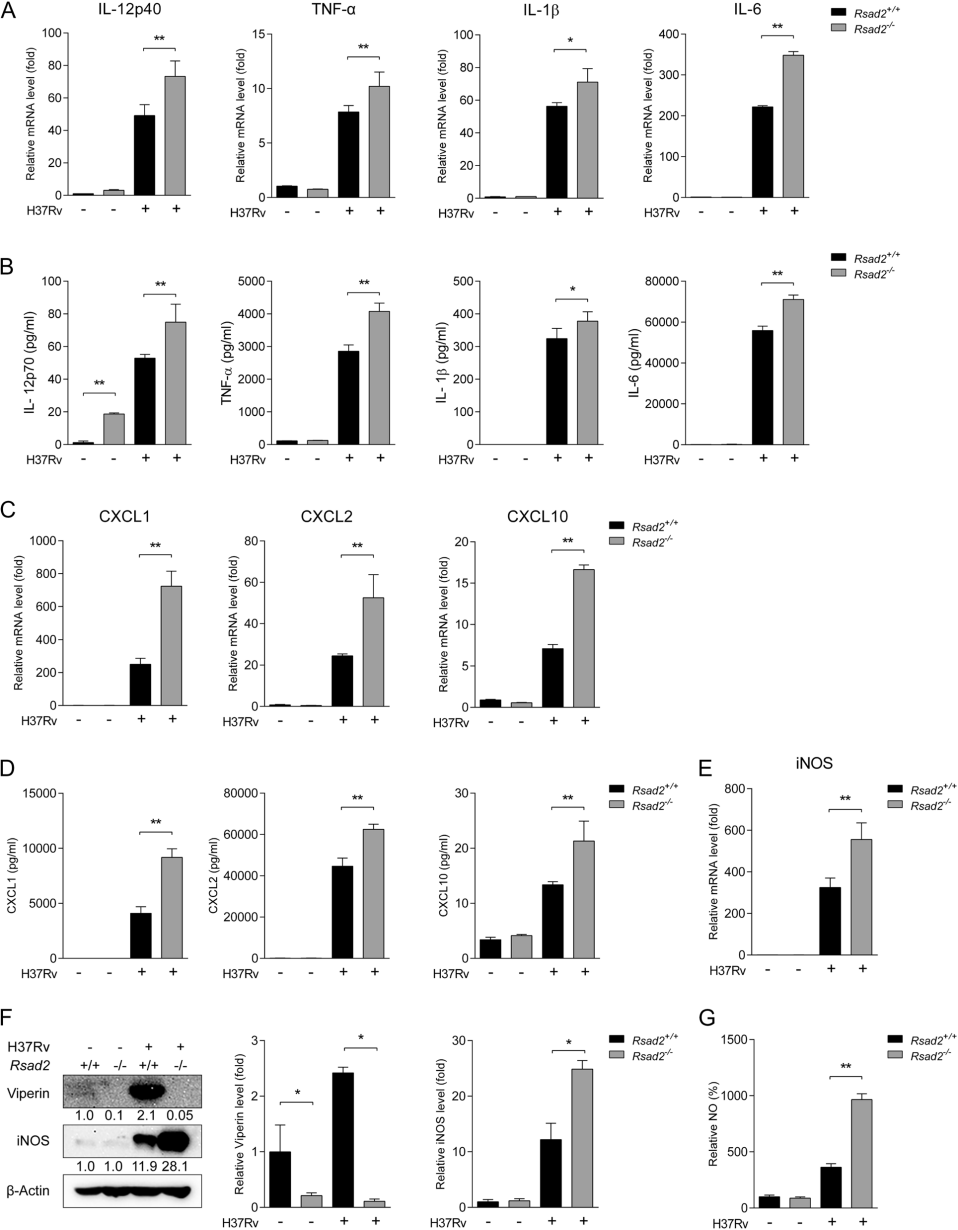
Viperin deficiency elicits robust cytokine, chemokine and NO production of DCs. *Rsad2*^*−/−*^ BMDCs were infected with H37Rv (MOI = 2) for 24 h. **A** Proinflammatory cytokines including IL-12p40/IL-12p70, TNF-α, IL-1β and IL-6 mRNA levels were validated by qRT-PCR and **B** secretion levels were detected by ELISA. **C** Chemokines including CXCL1, CXCL2 and CXCL10 mRNA levels were validated by qRT-PCR and **D** secretion levels were detected by ELISA. **E** iNOS mRNA level was detected by qRT-PCR and **F** protein expression was detected by Western blot. **G** NO production was detected by Griess Reagent System. **A**, **C**, **E** Data are presented as fold change relative to uninfected controls of *Rsad2*^+*/*+^ BMDCs and at least three independent experiments, with each 2–3 replicates. **B**, **D** Data are at least n = 3 independent experiments with each 2–3 replicates are shown. **F** Densitometric analysis was performed after Western blot analysis. β-Actin served as an internal reference. The numbers below immunoblot indicates the density ratios of (Viperin or iNOS)/β-Actin. Data are presented as fold change relative to uninfected controls of *Rsad2*^+*/*+^ BMDCs and are representative of three independent experiments with similar results. The ratios of protein expressions of viperin and iNOS are shown in graph. **G** Data are presented with the uninfected control of *Rsad2*^+*/*+^ BMDCs is used as 100%. **A**–**G** Data shown are the mean ± SD, *T*-test, *p ≤ 0.05, **p ≤ 0.01

A subset of proinflammatory cytokines and chemokines produced by DCs play vital roles in recruiting and activating different immune cells to the site of Mtb infection. We detected cytokine and chemokine mRNA abundance by qRT-PCR and validated the production by ELISA assay. We obtained that cytokines including IL-12p40/IL-12p70, TNF-α, IL-1β and IL-6 were significantly increased in *Rsad2*^−/−^ BMDCs compared with those in *Rsad2*^+/+^ BMDCs (Fig. 3A and B). Chemokines including CXCL1, CXCL2 and CXCL10 production were significantly increased in Viperin deficient BMDCs (Fig. 3C and D). However, the production of cytokine TGF-β and chemokines of CCL3 and CCL4 were not influenced in *Rsad2*^−/−^ BMDCs with Mtb infection (Supplementary Fig. 1A and B). Cholesterol accumulation, metabolic process and autophagy have been reported that contribute to manipulating immune responses against Mtb infection in DCs [14–17]. Here, however, cholesterol production, expression of important enzymes in the glycolytic pathway including SLC2α, PFKFB3, HK2, PDK1, HIF-1α, TKT or autophagy were not affected in *Rsad2*^−/−^ BMDCs with Mtb infection (Supplementary Fig. 1C–E). Reactive oxygen/nitrogen species and NO production are critical host defense pathways in determining the fate of intracellular Mtb within phagocytes. We further investigated whether Viperin deficiency influences iNOS expression and NO production. We demonstrated that mRNA and protein abundance of iNOS were significantly increased upon Mtb infection in *Rsad2*^−/−^ BMDCs (Fig. 3E and F). Consistently, NO production was observed with significant increase (Fig. 3G). These results suggest that Viperin deficiency elicits robust cytokine, chemokine and NO production of DCs.

### Viperin deficiency enhances expression of MHC I, II and co-stimulatory molecules in Mtb infected-DCs

**Fig. 4:**
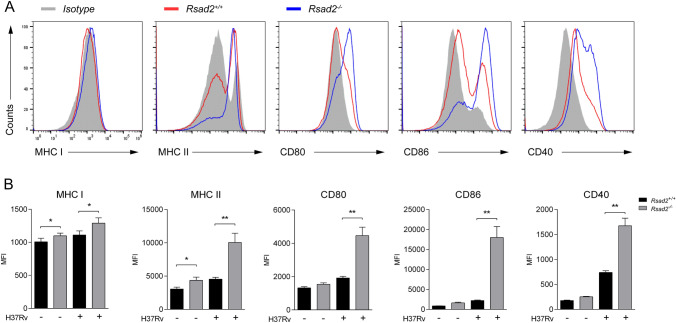
Viperin deficiency enhances expression of MHC I, MHC II and co-stimulatory molecules in Mtb infected-DCs. *Rsad2*^*−/−*^ BMDCs infected with H37Rv (MOI = 2) for 24 h. **A**, **B** The expressions of MHC I, MHC II, CD80, CD86 and CD40 on BMDCs (CD11c^+^) were detected via flow cytometry and MFI were assessed. Data are representative of three independent experiments with each 4 replicates. Data shown are the mean ± SD, *T*-test, *p ≤ 0.05, **p ≤ 0.01

MHC I, MHC II and co-stimulatory molecules, including CD80, CD86 and CD40, which are upregulated by Mtb infection, directly modulate the function of DCs in their antigen presentation and T cell immune responses against Mtb. We detected MHC I, II and co-stimulatory molecules of CD80, CD86 and CD40 expression by flow cytometry, and the results showed that expression of all these molecule were significantly enhanced in *Rsad2*^−/−^ BMDCs with Mtb infection for 24 h (Fig. 4A and B).

### Viperin deficiency promotes DC-mediated cross-priming of CD4^+^ and CD8^+^ T-cell activation

**Fig. 5:**
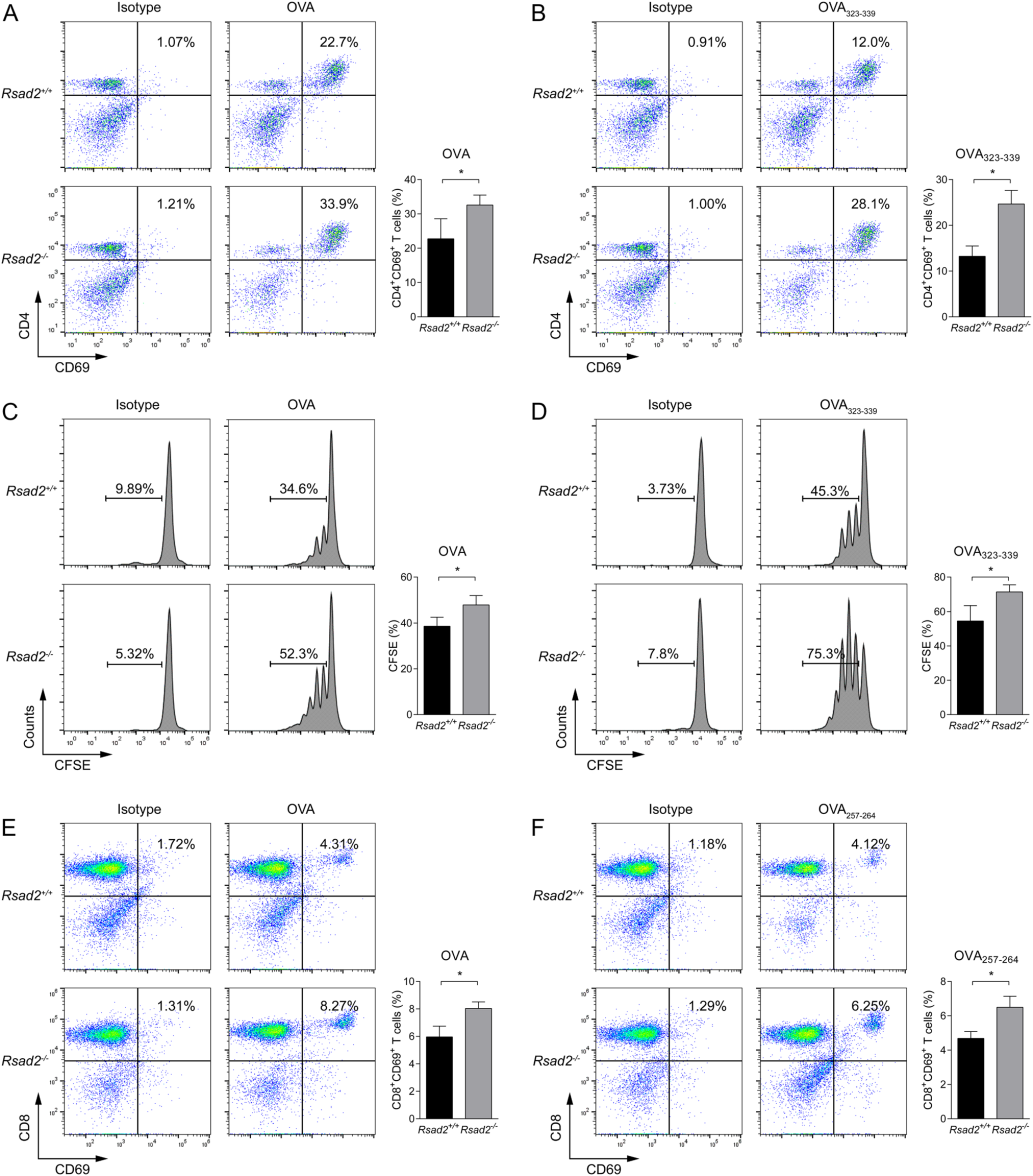
Viperin deficiency promotes DC function in T-cell activation. **A**, **B** CD4^+^ T cells from OT-II mice purified, BMDCs were incubated with OVA (1 mg/ml) or OVA_323–339_ (1 ug/ml) for 6 h and were co-cultured with purified T cells at a ratio of DC: T cell = 1: 10. Expression of CD69 was detected on CD4^+^ T cells via flow cytometry at 24 h. **C**, **D** CD4^+^ T cells from OT-II mice were purified and CFSE were stained, BMDCs were incubated with OVA (1 mg/ml) or OVA_323–339_ (1 ug/ml) for 6 h and were co-cultured with purified T cells at a ratio of DC: T cell = 1: 10. CD4^+^ T cells proliferation was determined by detecting the fluorescence intensity of CFSE via flow cytometry at 72 h. **E**, **F** CD8^+^ T cells from OT-I mice purified, BMDCs were incubated with OVA (1 mg/ml) or OVA_257–264_ (1 ug/ml) for 6 h and were co-cultured with purified T cells at a ratio of DC: T cell = 1: 10. Expression of CD69 was detected on CD8^+^ T cells via flow cytometry at 24 h. **A**–**F** Pooled data are presented in the right panel. Data are representative of 3 independent experiments with each 4 replicates. Data shown are the mean ± SD, *T*-test, *p ≤ 0.05

To investigate how Viperin regulates T cell responses, we co-cultured DCs with naïve ovalbumin-specific TCR transgenic CD4^+^ T cells (OT-II) for 6 h in the presence of OVA or cognate peptide (OVA_323–339_). The results showed that Viperin deficiency significantly increased the proportion of CD4^+^ CD69^+^ T cells (Fig. 5A and B). Next, we used fluorescent tag CFSE to detect cell proliferation. Fluorescent tags can be evenly distributed to two daughter cells during cell proliferation and its fluorescence intensity is half of parental cells. CD4^+^ T cells were sorted and co-cultured with BMDCs under the stimulation of OVA or OVA_323–339_ for 72 h. CD4^+^ T cells were collected and detected for the fluorescence intensity of CFSE by flow cytometry. We observed that Viperin deficiency led to decrease the fluorescence intensity of CFSE, indicating higher proliferation ability of CD4^+^ T cells (Fig. 5C and D). Then, we co-cultured DCs with naïve ovalbumin-specific TCR transgenic CD8^+^ T cells (OT-I) for 6 h in the presence of OVA or cognate peptide (OVA_257–264_). We found that Viperin deficiency significantly increased the proportion of CD8^+^ CD69^+^ T cells (Fig. 5E and F). We conclude that Viperin inhibits the antigen presentation ability of BMDCs, which subsequently inhibits T cell activation and proliferation.

### Viperin regulates DC function though activating NF-κB p65

**Fig. 6:**
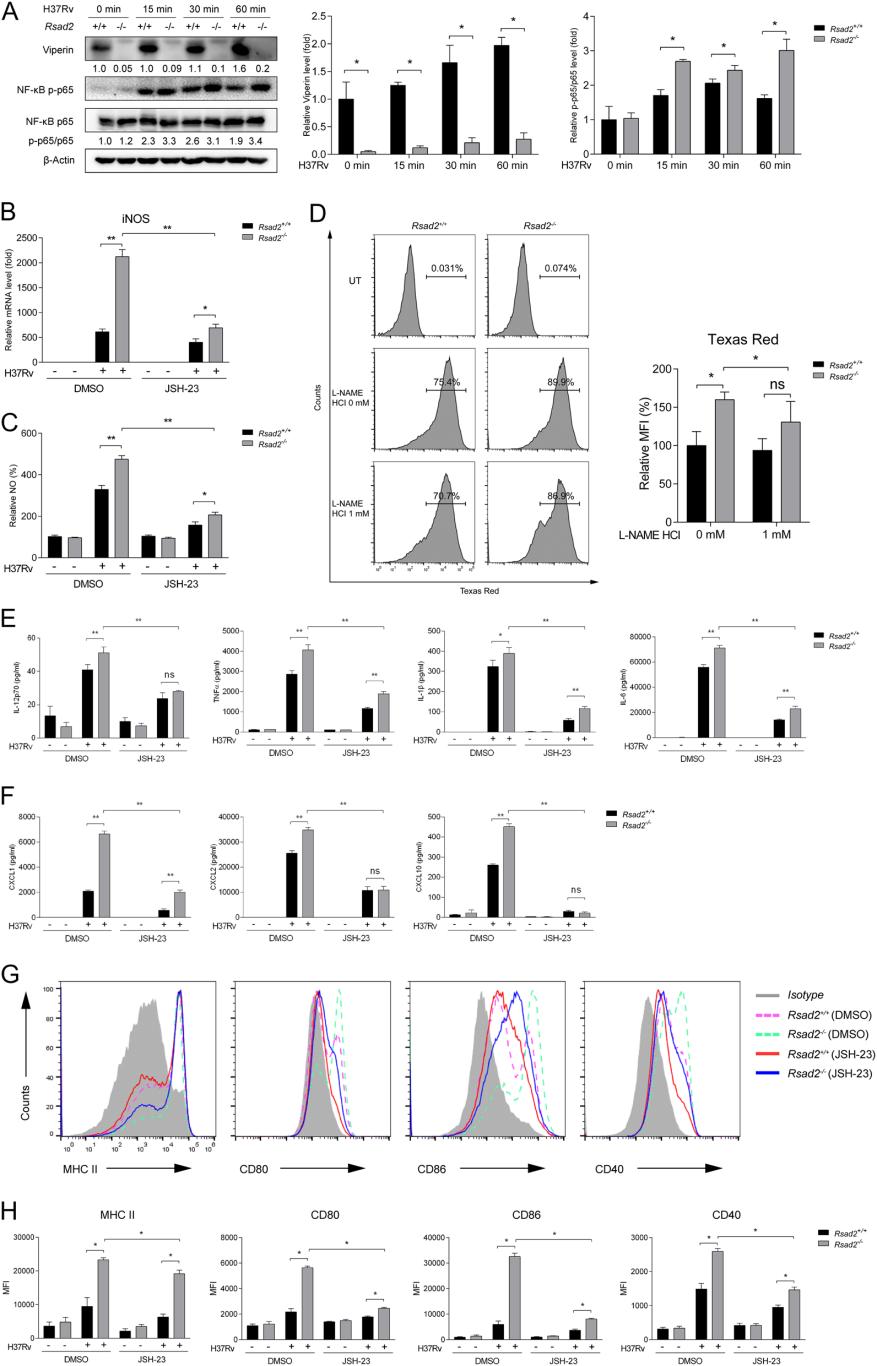
Viperin regulates DC function though activation of NF-κB p65 signaling. **A** Phosphorylation level of NF-κB p65 was detected by Western blot in *Rsad2*^*−/−*^ BMDCs infected with H37Rv (MOI = 5) for 15, 30 and 60 min. **B**, **C**, **E**–**H** *Rsad2*^+*/*+^ BMDCs and *Rsad2*^*−/−*^ BMDCs were pretreated with JSH-23 (20 uM) for 1 h, following H37Rv infection (MOI = 2) for 24 h. **B** iNOS mRNA level was detected by qRT-PCR. **C** NO production was detected by Griess Reagent System. **D** *Rsad2*^+*/*+^ and *Rsad2*^*−/−*^ BMDCs were pretreated with L-NAME HCI (1 mM) for 1 h, following Texas Red tagged H37Rv infection for 2 h at MOI = 5. Cells were collected and incubated with fluorescently conjugated mAb CD11c^+^ at 4 °C for 30 min. Percentage of Texas Red positive BMDCs (CD11c^+^) and MFI were analyzed by flow cytometry. **E** Proinflammatory cytokines including IL-12p70, TNF-α, IL-1β, and IL-6 and **F** chemokines including CXCL1, CXCL2, and CXCL10 secretion levels were detected by ELISA**. G**, **H** The expressions of MHC II, CD80, CD86 and CD40 on BMDCs (CD11c^+^) were detected by flow cytometry and MFI were assessed. **A** Densitometric analysis was performed after Western blot analysis. β-Actin served as an internal reference. The numbers below immunoblot indicate the density ratios of (Viperin or p-p65/p65)/β-Actin. Data are presented as fold change relative to uninfected controls of *Rsad2*^+*/*+^ BMDCs and at least *n* = 3 independent experiments with each 2 replicates are shown. The ratios of protein expressions of Viperin and p-p65/p65 were shown in GAPDH. **B** Data are presented as fold change relative to uninfected control of *Rsad2*^+*/*+^ BMDCs and at least *n* = 3 independent experiments with each 2 replicates are shown. **C** Data are presented with the uninfected control of *Rsad2*^+*/*+^ BMDCs is used as 100% and at least three independent experiments, with each 2–3 replicates. **D** Data are presented with the 0 mM control of *Rsad2*^+*/*+^ BMDCs is used as 100% and are representative of three independent experiments with each 4 replicates. **E**, **F** Data are at least *n* = 3 independent experiments with each 2 replicates are shown. **G**, **H** Data are representative of three independent experiments with each 4 replicates. **A**–**H** Data shown are the mean ± SD, *T*-test, *p ≤ 0.05, **p ≤ 0.01, ns (not significant)

It is reported that Viperin was associated with both IRAK1 and TRAF6 and activated downstream signaling. To further investigate the exact mechanisms of Viperin in regulating DC functions, we detected TAK1, IKKα/β, MAPKs and NF-κB activation by Western blot analysis. The results showed that phosphorylation of TAK1, IKKα/β, MAPKs SAPK/JNK, ERK1/2 and p38 were not affected by Viperin deficiency after Mtb infection for 15, 30 and 60 min (Supplementary Fig. 2A). NF-κB p65 phosphorylation was significantly increased in *Rsad2*^−/−^ BMDCs compared with *Rsad2*^+/+^ BMDCs with Mtb infection (Fig. 6A). Next, we applied NF-κB p65 inhibitor JSH-23 in *Rsad2*^−/−^ BMDCs, and found that Viperin deficiency induced iNOS expression and NO production were impaired by JSH-23 treatment (Fig. 6B and) C). NO inhibitor L-NAME HCl treatment inhibited Viperin deficiency induced phagocytosis of Mtb within DCs (Fig. 6D), but did not influence MHC II, CD80 and CD86 expression (Supplementary Fig. 2B). These results indicated that Viperin could suppress phagocytic function of DCs via NF-κB p65 activation and NO production. Moreover, JSH-23 treatment significantly repressed cytokine production of IL-12, TNF-α, IL-1β and IL-6 (Fig. 6E). Similarly, chemokine production including CXCL1, CXCL2 and CXCL10 expression was significantly reduced by JSH-23 treatment in *Rsad2*^−/−^ BMDCs (Fig. 6F). Importantly, Viperin deficiency promoted MHC II, CD80, CD86 and CD40 expression was inhibited by JSH-23 treatment in *Rsad2*^−/−^ BMDCs (Fig. 6G and H). Together, these results indicated that Viperin suppresses DC functions though NF-κB p65.

## Discussion

DCs are critical for initiation of adaptive immune responses to eliminate invading Mtb. In this study, we demonstrated that Viperin deficiency DCs showed robust activation and enhanced CD4^+^ and CD8^+^ T cell responses. We have shown that Viperin deficient DCs produced higher levels of NO, cytokines and chemokines expression, elevated co-stimulatory molecules expression, and enhanced CD4^+^ and CD8^+^ T cell responses. These data suggest that Viperin deficiency could boost the immune responses of DCs during Mtb infection, and we propose it constituting a potential target for future HDT in TB therapy.

IFNs are originally identified on the basis of their antiviral effects. Recent studies have highlighted the production of IFNs by Mtb infection in macrophages and DCs [18]. Although production of IFNs in infected cells serves as a pivotal event in innate immune response, these cytokines modulate the proliferation, maturation, differentiation and activation of DCs, NK, Th1 and memory CD8^+^ T cells [19, 20]. IFNs can also link innate and adaptive immune response by activating DCs. However, little is known about how interferon stimulated genes (ISGs) function on different lymphocytes. Viperin is one of important ISGs that can regulate the infection by directly interacting with viral protein or modulating the immune response of macrophages, DCs and T cells. Viperin deficiency facilitates polarization of BMDMs into M1 and M2 macrophages and secretion of cytokines [21]. Viperin can be induced by IRF-3 and IRF-7 mediated production of IFNs and combat different virus infection in myeloid DCs [22, 23]. In one study, Viperin was induced by dsRNA and LPS, and facilitated TLR7- and TLR9-mediated production of type I IFNs in plasmacytoid DCs (pDCs) [11]. Similarly, another study reported that Viperin was upregulated in fully matured DCs, which promoted production of IFN-I and secretion of pro-inflammatory cytokines, leading to enhanced T cell proliferation for antitumor response [12]. Viperin promoted T cell receptor-induced GATA-3 activation and optimal Th2 cytokine production via NF-κB and AP-1 activation [13]. In this study, Mtb infection facilitated Viperin expression in monocyte derived DCs. These results suggest that Viperin might play a role in regulating immune response against Mtb infection. However, we demonstrated that during Mtb infection, Viperin suppressed the activation of DCs. This discrepancy of Viperin on functions of DCs might be due to various models and different stimulus sources. Although IFNs are essential for the activation of adaptive immune responses through modulating DC activity [24], several reports underline that high levels of IFNs played an opposite role in combating Mtb [25]. For example, IFNs could act as negative host factors under the control of IL-1 triggered PGE2 synthesis to determine the outcome of TB [26]. It suggests that IFNs, especially its down-stream ISGs could play different roles in immune responses against Mtb infection. Furthermore, Mtb has evolved tools and strategies to hijack the innate immune system, such as cytosolic escape, block of phagosome maturation, apoptosis, inflammasome activation/modulation as well as autophagy inhibition. As Viperin expression has been upregulated by Mtb infection (Fig. 1), identifying the role of Viperin in controlling DCs activation and function during Mtb infection is crucial for better understanding of immune response and will also have important clinical implications for TB treatment.

It is remarkable that NO production in macrophages is a major effector for anti-mycobacterial action in both experimental and human TB [5]. The phagocytosis of Mtb by DCs is important for host defense [4]. How Viperin regulates NO production and Mtb infection in DCs needs further investigation. Moreover, the high-output expression of NO plays a key role in host defenses against intracellular Mtb [5]. It is reported that DC-derived NO controls the balance of effector and regulatory DC differentiation [27]. Specific inhibitors of NO prevented human DC maturation, with decreased expression of MHC class II, costimulatory and CD83 molecules and reduced IL-12 production [28]. Recently, autophagy gains increased attention on its ability of manipulating immune responses against Mtb infection especially in DCs [14]. Although autophagy might contribute to antigen processing for MHC II presentation, we found that Viperin did not affect autophagy in Mtb-infected DCs. Cholesterol accumulation in the cell membrane of DCs enhanced MHC II dependent antigen presentation and CD4^+^ T cell activation [15, 16]. Metabolic processes and their molecular signaling pathways have the effect on DC development and differentiation [17]. It is reported that Viperin could inhibit virus infection via reduced cholesterol biosynthesis or improve glucose metabolism [29–31]. However, in this study, Viperin did not influence cholesterol production or gene expression of important enzymes in the glycolytic pathway including SLC2α, PFKFB3, HK2, PDK1, HIF-1α and TKT (Supplementary Fig. 1). Activation DCs by Mtb infection upregulates MHC I, II and co-stimulated factors of CD80, CD86 and CD40 expression, and increases production of proinflammatory cytokines such as IL-12, TNF-α, IL-1β and IL-6, which promotes antigen processing and presentation [6, 7]. Mtb-infected DCs produce chemokines orchestrating the recruitment of cells into the Mtb-infected lung and contribute to Mtb containment [32]. For example, mature DCs could produce CCL3 and CCL4 after Mtb infection, which further recruit immature DCs to the site of infection and replace the mobilized population. Mtb-infected mature DCs express several chemokines such as CCL3, CCL4 and CXCL10 to stimulate NK and T cell migration [18]. Mtb-induced CXCL1 and CXCL2 could play important roles in recruiting neutrophils to sites of infection and control the early infection [33]. Innate immunity initially predominates in anti-Mtb responses, the subsequent recruitment and activation of T lymphocytes is indispensable for combating Mtb. Most of the reports on the effects of Viperin were related to how it regulates the viral infection. However, some bacteria such as *Shigella flexneri* and *Listeria monocytogenes* could induce Viperin expression and Viperin has been shown to inhibit these bacterial infections by promoting type I interferon response. Viperin deficiency promoted cellular cholesterol and *Shigella flexneri* entry into cells. This study first highlighted the role of Viperin and the type I IFN response in the control of bacterial pathogens [34, 35]. In our study, we highlight that targeting Viperin could be a potential strategy to enhance DC-mediated immune response against Mtb infection.

Several researches have focused on the mechanisms of Viperin in regulating immune responses. Viperin could catalyze the conversion of cytidine triphosphate (CTP) to 3’-deoxy-3’,4’-didehydro-CTP (ddhCTP), and act as a chain terminator of the RNA-dependent RNA-polymerases from many viruses, via a SAM-dependent radical way [36]. It could also regulate other innate immune responses by binding MAVS and trigger macrophage polarization [21, 37]. In addition, Viperin has been demonstrated as a host factor to couple innate immune signaling to antiviral ribonucleotide synthesis by interacting with the kinase IRAK1 and the E3 ubiquitin ligase TRAF6 [38]. Another study reported that Viperin facilitated IRF-7 mediated IFN-I production and T cell proliferation to induce antitumor response, however, without affecting NF-κB p65 activation [12]. In this study, we found that Viperin did not influence TAK1, IKKα/β and MAPKs signaling activation, which are downstream factors of IRAK1 and TRAF6. However, Viperin deficiency activated NF-κB p65 signaling. Activation of NF-κB signaling in DC, triggered by either pathogen-associated molecular patterns or proinflammatory cytokines, induces most of the typical phenotypic and functional characteristics of mature DCs, inducing the expression of MHC II and co-stimulatory molecules [39–41]. In this study, we demonstrated that Viperin could inhibit phagocytosis of Mtb via NO production induced by NF-κB p65 phosphorylation. Furthermore, Viperin suppressed cytokine including IL-12, TNF-α, IL-1β, IL-6 and chemokine including CXCL1, CXCL2, CXCL10 production and co-stimulated factor expression of DCs during Mtb infection via inhibiting NF-κB p65 activation (Fig. 6). These results suggest that NF-κB p65 activation plays a key role in Viperin inhibiting DC activation and function during Mtb infection. However, the exact mechanistic details of Viperin interaction with NF-κB signaling require further investigation.

## Conclusions

**Fig. 7:**
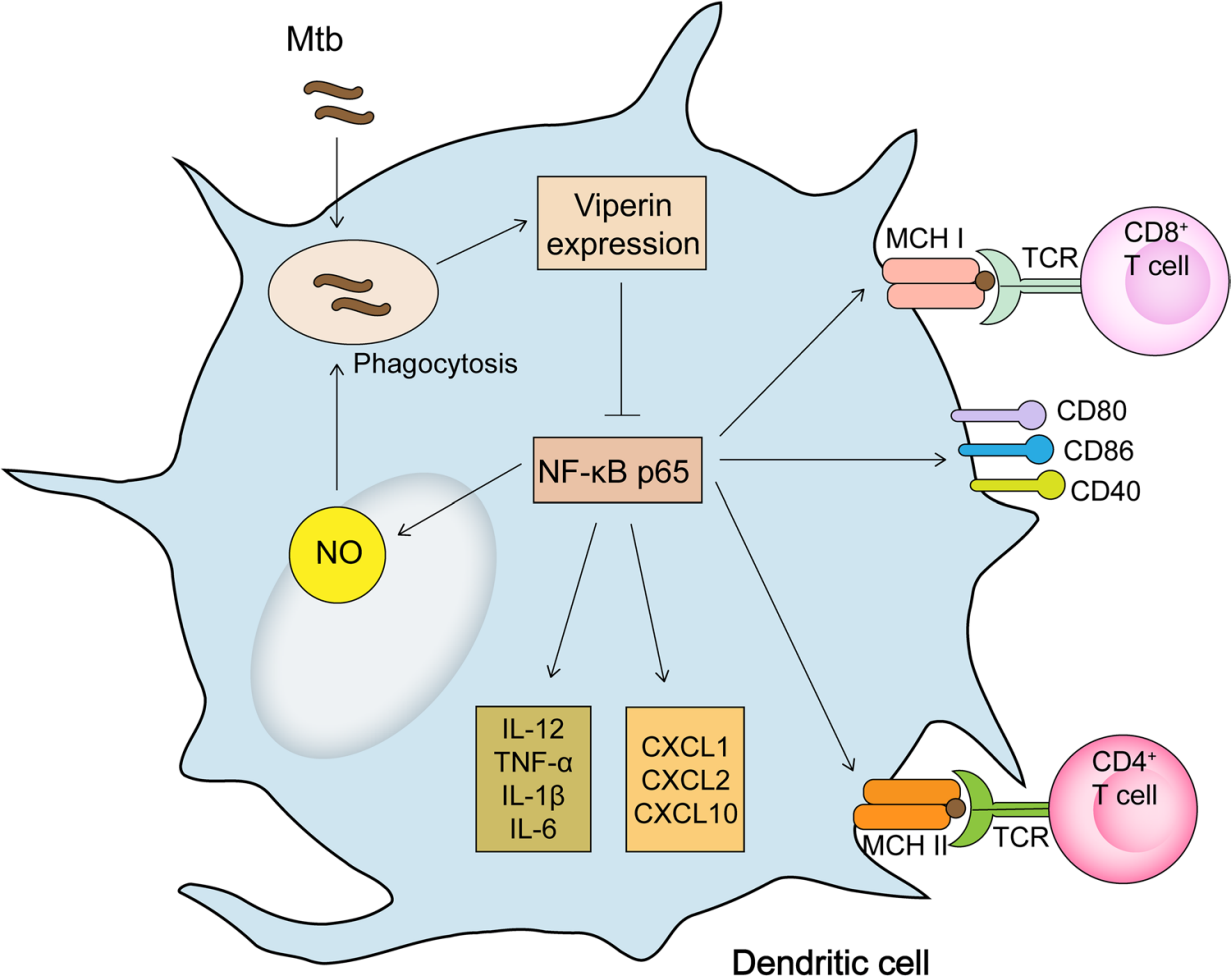
Illustration of the effects of Viperin on dendritic cell activation and function via NF-κB activation during Mtb infection

Given the role of DCs in anti-Mtb immunity, we sought to characterize the effects and mechanisms of Viperin on activating DCs during Mtb infection. In this study, we conclude that Viperin deficiency inhibits phagocytosis of Mtb, promotes clearance of Mtb in DCs, upregulates NO, cytokine and chemokine production, increases expression of MHC I, II and co-stimulatory molecules, and enhances DC function in promoting CD4^+^ and CD8^+^ T-cell activation (Fig. 7). We propose Viperin as a candidate target for adjunct HDT in TB, though, opposite effects of Viperin on promoting immune responses were observed in different experimental models. A better understanding and further investigation of Viperin may develop new strategies against Mtb infection and improve current treatment for TB patients.

## Supplementary Information

Below is the link to the electronic supplementary material.

Supplementary file1 (DOCX 572 KB)

## Data Availability

All data generated or analyzed during this study are included in this article [and/or] its supplementary material files. Further enquiries can be directed to the corresponding author.
