# Supplementary material for: Viperin deficiency promotes dendritic cell activation and function via NF-kappaB activation during Mycobacterium tuberculosis infection
Source: Inflamm Res. 2022 Oct 31;72(1):27–41. doi: 10.1007/s00011-022-01638-3 (PMC9902321; doi:10.1007/s00011-022-01638-3)
Supplement: Supplementary file 1 — Supplementary file1 (DOCX 572 KB) [file 11_2022_1638_MOESM1_ESM.docx]

**Viperin deficiency promotes dendritic cell activation and function via NF-kappaB activation during *Mycobacterium tuberculosis* infection**

**Short titles:** Viperin in Mtb-infected DCs

Xinying Zhou^¶*^, Hui Xu^¶^, Qianna Li, Qi Wang, Honglin Liu, Yingqi Huang, Yao Liang, Linmiao Lie, Zhenyu Han, Yaoxin Chen, Yulan Huang,Wenle Zhou, Qian Wen, Chaoying Zhou, Shengfeng Hu, Li Ma^*^

Institute of Molecular Immunology, School of Laboratory Medicine and Biotechnology, Southern Medical University, Guangzhou 510515, China.

^¶^Both authors contributed equally.

*Corresponding authors:

Dr. Li Ma

Li Ma, PhD, Institute of Molecular Immunology, School of Laboratory Medicine and Biotechnology, Southern Medical University, Guangzhou 510515, China. Phone: +86-20-61648322, Fax: +86-20-61648322. E-mail: mali_61648322@smu.edu.cn

Dr. Xinying Zhou

Xinying Zhou, PhD, Institute of Molecular Immunology, School of Laboratory Medicine and Biotechnology, Southern Medical University, Guangzhou 510515, China. Phone: +86-20-61648553, Fax: +86-20-61648322. E-mail: zxyforever@smu.edu.cn

**SUPPLEMENTARY MATERIAL**

**Figures S1-2**

**Tables S1**

**
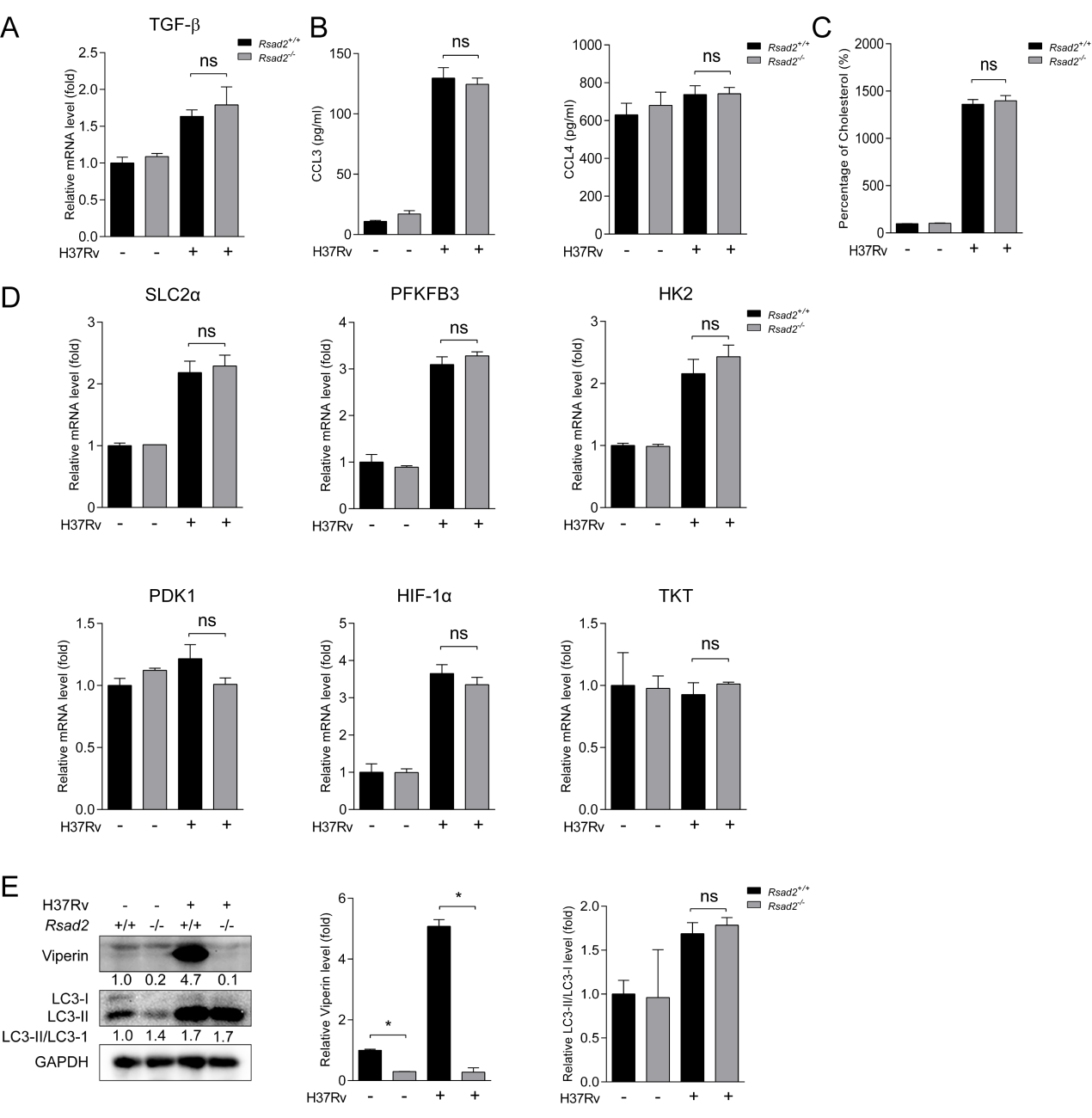
**

**Supplementary Fig. 1 Viperin deficiency did not influence TGF-β, CCL3 and CCL4 expression, cholesterol production, expression of important enzyme involved in the glycolytic pathway including SLC2α, PFKFB3, HK2, PDK1, HIF-1α, TKT or autophagy in Mtb-infected DCs.** *Rsad2^+/+^* BMDCs and *Rsad2^-/-^* BMDCs infected with H37Rv (MOI=2) for 24 hrs. **(A, D)** TGF-β and SLC2α, PFKFB3, HK2, PDK1, HIF-1α, TKT mRNA levels were validated by qRT-PCR. (B) CCL3 and CCL4 secretion levels were detected by ELISA. (C) Cellular cholesterol was detected by Amplite™ Cholesterol Quantitation Kit. (E) LC3 protein expression was detected by Western blot. **(A, D)** Data are presented as fold change relative to uninfected controls of *Rsad2^+/+^* BMDCs. Data are at least three independent experiments, with each 2-3 replicates. (B) Data are at least three independent experiments, with each 2-3 replicates. (C) Percentage of cholesterol was normalized by cellular cholesterol in uninfected *Rsad2^+/+^* BMDCs. Data are at least three independent experiments, with each 2-3 replicates. (E) Densitometric analysis was performed after Western blot analysis. GAPDH served as an internal reference. The numbers below immunoblot indicates the density ratio of (Viperin or LC3-I/LC3-II)/GAPDH. Data are presented as fold change relative to uninfected controls of *Rsad2^+/+^* BMDCs and at least n = 3 independent experiments with each 2 replicates are shown. The ratios of protein expressions of Viperin and LC3-I/LC3-II are shown in graph.

**(A-E)** Data shown are the mean ± SD, T-test, *p ≤ 0.05, ns (not significant).

**
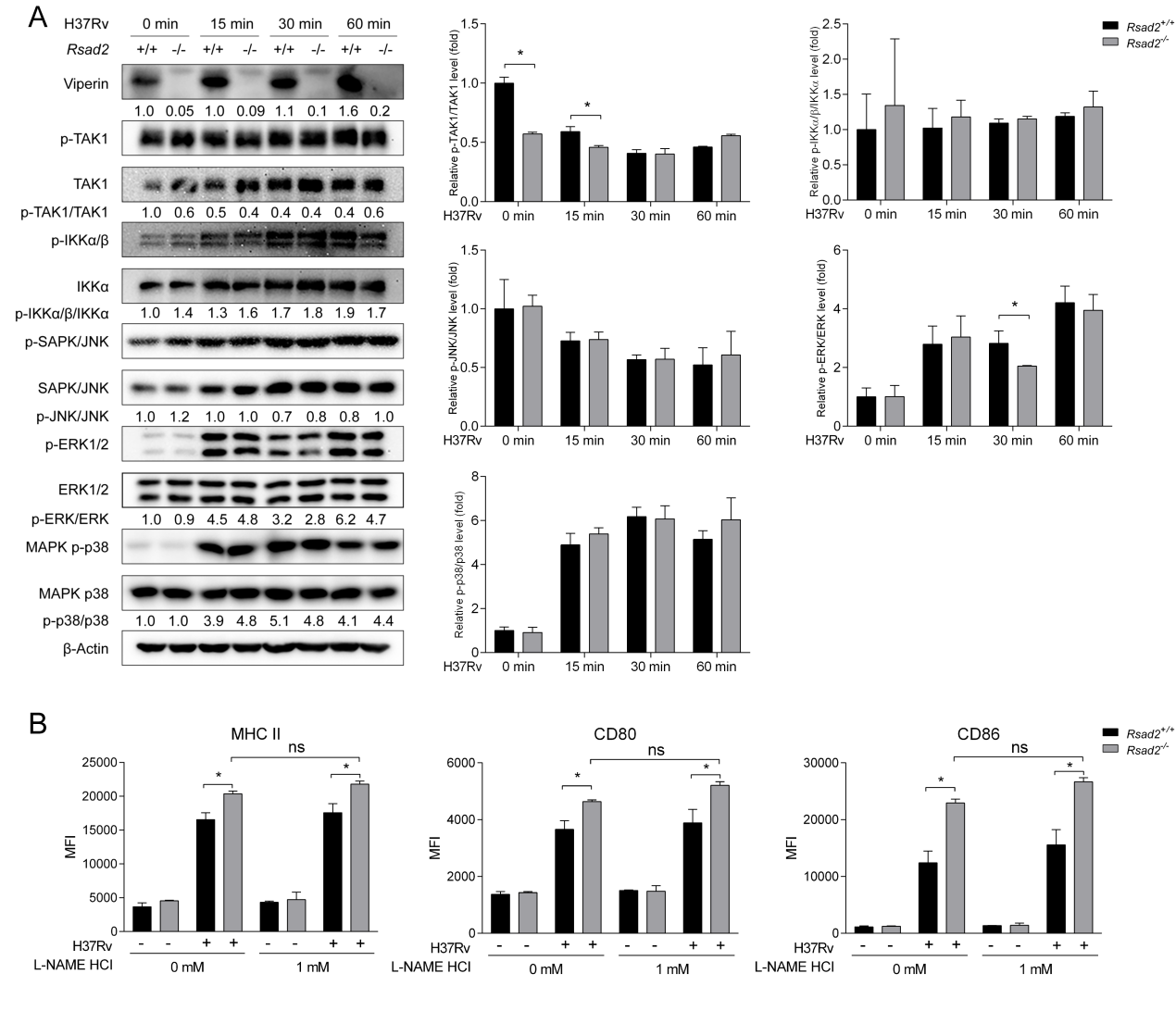
**

**Supplementary Fig. 2 Viperin deficiency did not influenced phosphorylation of TAK1, IKKα/β, MAPKs SAPK/JNK, ERK1/2 and p38 and L-NAME HCl treatment did not influence MHC II, CD80 and CD86 expression.**

(A) The kinase phosphorylation levels of TAK1, IKKα/β, MAPKs SAPK/JNK, ERK1/2 and p38 signaling pathways were detected by Western blot in *Rsad2^-/-^* BMDCs infected with H37Rv (MOI=5) for 15, 30 and 60 min. (B) BMDCs were pretreated with L-NAME HCI (1mM) for 1 hr, following Texas Red tagged H37Rv infection for 24 hrs at MOI=2. The expressions of MHC II, CD80 and CD86 on BMDCs (CD11c^+^) were assessed by MFI via flow cytometry. (A) Densitometric analysis was performed after Western blot analysis. β-Actin served as an internal reference. The numbers below immunoblot indicates the density ratios of (Viperin or target phosphorylated protein/total protein)/β-Actin. Data were presented as fold change relative to uninfected controls of *Rsad2^+/+^* BMDCs and at least n = 3 independent experiments with each 2 replicates are shown. The ratios of protein expressions of Viperin and target phosphorylated protein/total protein are shown in graph. (B) Data are presented as fold change relative to uninfected controls of *Rsad2^+/+^* BMDCs and at least n = 3 independent experiments with each 2 replicates are shown. (A, B) Data shown are the mean ± SD, T-test, *p ≤ 0.05, ns (not significant).

**Table S1. List of antibodies.**

| **Antibodies** | **Application** | **Dosage** | **source** |
| --- | --- | --- | --- |
| GAPDH | WB | 1:1000 | ZSGB-BIO 17AF0412 |
| β-actin | WB | 1:1000 | CST #8457 |
| Viperin [MaP.VIP] | WB | 1:50 | Abcam ab107359 |
| iNOS | WB | 1:1000 | CST #13120S |
| Phospho-NF-κB p65 (Ser536) | WB | 1:1000 | CST #3033S |
| NF-κB p65 | WB | 1:1000 | CST #8242S |
| Phospho-TAK1 (Ser412) | WB | 1:1000 | CST #9339 |
| TAK1 | WB | 1:1000 | CST #5206 |
| Phospho-IKKα/β (Ser176/180) | WB | 1:1000 | CST #2697S |
| IKKα | WB | 1:1000 | CST #2682S |
| Phospho-SAPK/JNK (Thr183/Tyr185) | WB | 1:1000 | CST #4370S |
| SAPK/JNK | WB | 1:1000 | CST #9252S |
| Phosphop44/42MAPK (Erk1/2)(Thr202/Tyr204) | WB | 1:1000 | CST #4370S |
| p44/42 MAPK (Erk1/2) | WB | 1:1000 | CST #4695S |
| Phospho-p38 MAPK (Thr180/Tyr182) | WB | 1:1000 | CST #4511S |
| p38 MAPK | WB | 1:1000 | CST #8690S |
| Goat anti-Mouse IgG (H+L) Secondary Antibody, HRP | WB | 1:3000 | Thermo 31430 |
| Goat anti-Rabbit IgG (H+L) Secondary Antibody, HRP | WB | 1:3000 | Thermo 31460 |
| LC3 A/B | WB | 1:1000 | CST #12741 |
| GAPDH | WB | 1:1000 | ZSGB-BIO 17AF0412 |
| β-actin | WB | 1:1000 | CST #8457 |
| Viperin [MaP.VIP] | WB | 1:50 | Abcam ab107359 |
| iNOS | WB | 1:1000 | CST #13120S |
| Phospho-NF-κB p65 (Ser536) | WB | 1:1000 | CST #3033S |
| NF-κB p65 | WB | 1:1000 | CST #8242S |
| Phospho-TAK1 (Ser412) | WB | 1:1000 | CST #9339 |
| TAK1 | WB | 1:1000 | CST #5206 |
| Phospho-IKKα/β (Ser176/180) | WB | 1:1000 | CST #2697S |
| IKKα | WB | 1:1000 | CST #2682S |
| IKKβ | WB | 1:1000 | CST #8943 |
| Phospho-SAPK/JNK (Thr183/Tyr185) | WB | 1:1000 | CST #4370S |
| SAPK/JNK | WB | 1:1000 | CST #9252S |
| Phosphop44/42MAPK (Erk1/2)(Thr202/Tyr204) | WB | 1:1000 | CST #4370S |
| p44/42 MAPK (Erk1/2) | WB | 1:1000 | CST #4695S |
| Phospho-p38 MAPK (Thr180/Tyr182) | WB | 1:1000 | CST #4511S |
| p38 MAPK | WB | 1:1000 | CST #8690S |
| Goat anti-Mouse IgG (H+L) Secondary Antibody, HRP | WB | 1:3000 | Thermo 31430 |
| Goat anti-Rabbit IgG (H+L) Secondary Antibody, HRP | WB | 1:3000 | Thermo 31460 |
| LC3 A/B | WB | 1:1000 | CST #12741 |
| GAPDH | WB | 1:1000 | ZSGB-BIO 17AF0412 |
| β-actin | WB | 1:1000 | CST #8457 |
| Viperin [MaP.VIP] | WB | 1:50 | Abcam ab107359 |
| iNOS | WB | 1:1000 | CST #13120S |
| Phospho-NF-κB p65 (Ser536) | WB | 1:1000 | CST #3033S |
| NF-κB p65 | WB | 1:1000 | CST #8242S |
| Phospho-TAK1 (Ser412) | WB | 1:1000 | CST #9339 |
| TAK1 | WB | 1:1000 | CST #5206 |
| Phospho-IKKα/β (Ser176/180) | WB | 1:1000 | CST #2697S |
| IKKα | WB | 1:1000 | CST #2682S |
| Phospho-SAPK/JNK (Thr183/Tyr185) | WB | 1:1000 | CST #4370S |
| SAPK/JNK | WB | 1:1000 | CST #9252S |

WB, Western blot.
